# Synthesis and hygrothermal aging of polycarbonates containing a bisphenol fluorene moiety†

**DOI:** 10.1039/d4ra03378g

**Published:** 2024-06-03

**Authors:** Tao Lu, Wei Fang, Qian Zhou, Mengjuan Liu, Guozhang Wu

**Affiliations:** a Shanghai Key Laboratory of Advanced Polymeric Materials, School of Materials Science and Engineering, East China University of Science and Technology Shanghai 200237 China wgz@ecust.edu.cn

## Abstract

This study aims to synthesize a specific type of polycarbonate with high refractive index, low birefringence, and resistance to hygrothermal aging by copolymerizing 2,2′-bis(2-hydroxyethoxy)-1,1′-binaphthyl (BNE) with 9,9-bis[4-(2-hydroxyethoxy)phenyl]fluorene (BPEF). Comparative analysis revealed that the copolymer synthesized from BNE and BPEF demonstrated superior hydrolytic stability relative to the bisphenol A-based polycarbonate. This augmented stability can be attributed to the monomers' higher p*K*_a_ values, rendering acidic substances less capable of dissociating and thereby mitigating ester hydrolysis under hygrothermal conditions. Furthermore, the investigation probed into the phenomenon of physical aging in copolymerized polycarbonate when exposed to hygrothermal environments. It was discerned that the enthalpy loss, observable under both dry and hygrothermal conditions, could be linearly correlated with the difference between the aging temperature and the glass transition temperature (*T*_g_), signifying a close correlation between the magnitude of physical aging and *T*_g_. A lower *T*_g_ in the copolymerized polycarbonate led to more pronounced physical aging within the same timeframe, resulting in an augmentation of tensile strength and modulus, while higher *T*_g_ effectively mitigated the physical aging phenomenon.

## Abstract 2

This study aims to synthesize a specific type of polycarbonate with high refractive index, low birefringence, and resistance to hygrothermal aging.

## Graphical abstract

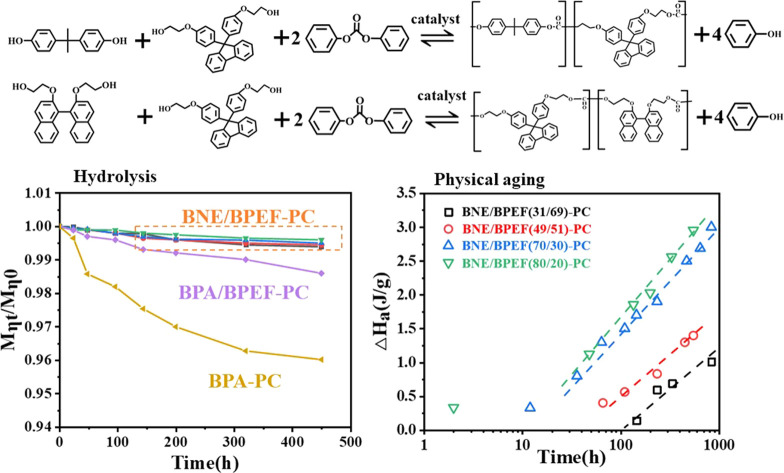

## Introduction

Bisphenol A polycarbonate (BPA-PC) is distinguished by its high strength, toughness, and optical clarity, rendering it indispensable in a multitude of optical applications, including lenses, glasses, and display screens. In the wake of rising interest in polymer-based ultra-thin optical lenses and smartphone lenses in recent years, a pivotal area of research has been the enhancement of the polymer's refractive index while concurrently minimizing birefringence. The polymer's refractive index is contingent upon the monomer's molar refractive index and its molar volume, with molecular chains featuring phenyl rings, naphthalene rings, and halogen heteroatoms typically exhibiting a higher molar refractive index.^1,2^ However, the interaction of light with phenyl ring structures alters light velocity, engendering significant birefringence.^3^ This interaction can precipitate light deviation and dispersion within the material, detracting from its transparency and optical quality. Given that polycarbonates with uniform structures usually manifest either positive or negative birefringence, it is imperative to employ monomers with positive birefringence in conjunction with those exhibiting negative birefringence through copolymerization to achieve reduced birefringence.^4^ It has been documented that bisphenol fluorene monomers with cardo structures not only elevate the refractive index but also mitigate birefringence owing to the four phenyl rings being located on different planes copolymerization with BPA, which has positive birefringence, can reduce the effect of birefringence.^5,6^ Du *et al.*^6^ copolymerized BPEF with BPA, confirming that the refractive index of the copolymer PC could vary between 1.586 and 1.639, and adjusting the copolymer ratio effectively reduced birefringence. Kato *et al.*^7^ synthesized high molecular weight and high refractive index BPEF-PC. However, it still fails to meet the requirements for a high refractive index. To further increase the refractive index, Li *et al.*^8^ introduced BNE and 9,9-bis(3-phenyl-4-hydroxyphenyl)fluorene for copolymerization, synthesizing a polycarbonate with a refractive index of up to 1.660.

Polycarbonate molecular chains are inherently sensitive to water due to the high polarity of carbonate bonds, rendering them susceptible to hydrolytic degradation.^9,10^ It is necessary to conduct hygrothermal aging assessments.^11,12^ Yan *et al.*^13^ investigated the hydrolysis mechanism of BPA-PC, revealing that an abundance of terminal hydroxyl groups exacerbates hydrolysis, attributed to the hydroxyl groups' pronounced hydrophilicity, potentially increasing water absorption rates.^14,15^ After hygrothermal aging tests, samples generally became “brittle,” a result not solely of hydrolytic molecular weight reduction but also potential physical aging during testing.^16^ The appearance of physical aging is mainly because glassy polymers are always in metastable non-equilibrium state. Microscopic mobility leads to local conformational rearrangements within the molecular chains, allowing the polymer structure to relax towards a lower energy equilibrium state, manifesting macroscopically as changes in material density, modulus, and yield strength.^17,18^ It is noteworthy that physical aging only changes the stacking density of the molecular weight without causing chain breaks and can be reversed by thermal treatment above *T*_g_, hence it is reversible.^19^ Ohara *et al.*^20^ compared the differential scanning calorimetry (DSC) and mechanical tensile test results of BPA-PC before and after physical aging, finding a close correlation between enthalpy relaxation kinetics and yield stress changes.

This study employed the melt transesterification method^21,22^ to copolymerize BPEF with BPA and BNE respectively to synthesize PC copolymers. It meticulously evaluates the influence of monomer types and copolymer ratios on the products' *T*_g_, refractive index, and water absorption rate, elucidates the factors affecting hydrolysis and physical aging in hygrothermal environments, and investigates the correlation between hygrothermal aging and the mechanical properties of copolymer PCs. This research lays the groundwork for further refinement of the molecular structure and composition of bisphenol fluorene-containing PCs to achieve high refractive indices and low birefringence effects.

## Experimental

### Raw materials

2,2′-Bis(2-hydroxyethoxy)-1,1′-binaphthyl (BNE, 98.9%) and 9,9-bis[4-(2-hydroxyethoxy)phenyl]fluorene (BPEF, 99.57%) were purchased from Jiangsu Yongxing Chemical. 4,4′-Dihydroxydiphenylpropane (BPA, 99.8%) was provided by Aladdin. Diphenyl carbonate (DPC, 99.0%) was supplied by Zhetie Dafeng Chemical. Anhydrous ethanol (99.7%), dichloromethane (99.5%), and chloroform (99.0%) were purchased from Shanghai Titan and Shanghai Adamas, respectively. Deuterated chloroform (99.8%) was bought from Shanghai Blingwei Chemical.

### Synthesis of PC

**Scheme 1:**
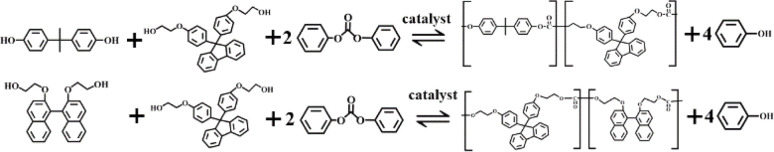
Reaction formula to synthesize the PC copolymers through the melt transesterification.

The copolymerization of BPEF with BPA and BNE was conducted using the melt transesterification method (Scheme 1). The reaction involved two stages under catalyst presence with DPC. Initially, the transesterification phase began following confirming that the reaction apparatus achieved an adequate vacuum level, accompanied by three successive purges of N_2_. Subsequently, 0.03523 mol of DPC and 0.03505 mol of dihydroxy monomers were then added under an N_2_ atmosphere, with a molar ratio of 1.005. The mixture was gradually heated to the predetermined transesterification temperature of 180 °C, after which a certain amount of catalyst was added, and stirring was initiated. The transesterification reaction proceeded for 60 min. Following this, the polycondensation stage aimed to gradually eliminate the byproduct phenol to drive the reaction forward. The procedure involved heating the mixture to 200 °C and applying a vacuum of 16 kPa for 20 minutes; elevating the temperature to 220 °C, meticulously reducing the pressure to 6 kPa, and maintaining the reaction for another 20 minutes; and, ultimately, increasing the temperature to 240 °C, applying a vacuum of 4 kPa, and reacting for 20 minutes. Afterward, the temperature was raised to 250 °C to maintain the vacuum below 80 Pa to remove residual phenol. The reaction ended after 30 min, the product was dissolved in dichloromethane, and the solution was slowly poured into ethanol to precipitate, which was then dried for further use.

### Preparation of PC films

Polycarbonate films were prepared by the solvent casting method. The samples were first dried in an oven at 80 °C for 12 h to remove moisture, then dissolved in dichloromethane to make a solution with a mass fraction of about 6%. The solution was degassed using ultrasonic oscillation, then cast onto a smooth surface dish and left at room temperature for 24 h to allow most of the solvent to evaporate. Finally, the samples were placed in a 30 °C oven and gradually heated in steps of 50, 70 and 90 °C to expel any residual solvent, each step maintained for 12 h. The samples were then heated above its *T*_g_ to remove thermal history, yielding a PC film with a thickness of 0.08 mm for subsequent water absorption and hygrothermal aging tests.

### Characterization

Molecular structure characterization was performed using ^1^H-NMR (400 MHz, Ascend 600, Bruker, Germany). Samples were dissolved in deuterated chloroform with tetramethylsilane as the internal standard. The calculation results of terminal hydroxyl groups ([–OH]) are shown in Table 1.

**Table 1:** Copolymerization ratio, viscosity-averaged molecular weight (*M*_*η*_), number-averaged molecular weight (*M*_n_, determined based on terminal groups measurements), color difference (Δ*C*), glass transition temperature (*T*_g_), light transmittance (*T*_580_), refractive index (*n*) and birefringence (Δ*n*)

		Mole ratio	*M*_*η*_ (kg mol^−1^)	*M*_n_ (kg mol^−1^)	[–OH] mol%	[–OPh] mol%	Δ*C* (%)	*T*_g_^o^C	*T*_580_ (%)	*n*_589_	Δ*n*_589_^a^ (×10^−3^)
PC-1	BNE/BPEF-PC	0/100	27.4	19.1	2.81	2.05	0.55	155	84	1.639	−0.83
PC-2		31/69	29.2	20.3	2.45	1.92	0.73	143	85	1.647	−0.28
PC-3		49/51	40.9	23.9	1.85	1.76	0.87	134	84	1.654	—
PC-4		70/30	26.1	19.6	1.77	2.49	0.74	128	84	1.659	−0.02
PC-5		80/20	24.2	17.6	1.92	2.75	0.51	125	84	1.661	—
PC-6		90/10	19.6	10.1	3.22	0.80	0.50	123	84	1.663	+0.18
PC-7	BPA/BPEF-PC	50/50	38.2	17.6	2.31	1.75	0.66	154	83	1.612	—
PC-8	BPA-PC	100	34.2	15.6	1.74	1.50	0.95	149	84	1.586	—

a Partial data on the Δ*n* resulting from molecular orientation were not recorded in the literature.

The molar content of terminal phenoxyl groups ([–OPh]) was determined using HPLC (Shimadzu, LC-20AD) with methanol as the mobile phase and a flow rate of 1 ml min^−1^. A 0.02 g sample was dissolved in 2 ml of tetrahydrofuran, followed by the addition of 1.5 ml of 10 wt% KOH/CH_3_OH solution for alcoholysis for 2 h. Then, 0.5 ml of acetic acid was added for neutralization, and the mixture was left to stand for 1 h, resulting in an alcoholysis solution concentration of 5 mg ml^−1^. The content of phenol (PhOH) was measured from the sample, and [–OPh] was calculated in Table 1.

The intrinsic viscosity-averaged molecular weight, *M*_*η*_, was measured using an Ubbelohde viscometer. The polymer product was dissolved in chloroform to prepare a solution with a concentration of 0.01 g ml^−1^. The temperature of the water bath was maintained at 25 ± 0.5 °C. The intrinsic viscosity [*η*] was calculated by the following formula:1
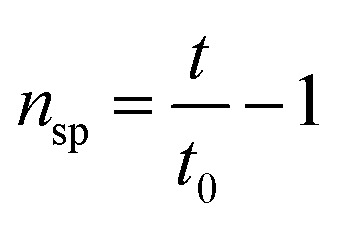
2
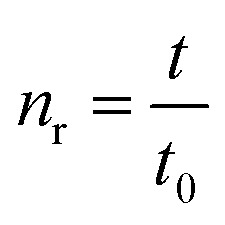
3
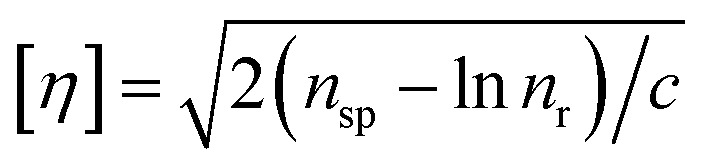
where *c* is the concentration of the prepared solution, *t*_0_ and *t* are the times it takes for the pure solvent and the sample solution to pass through two marks on the Ubbelohde viscometer, respectively. *η*_r_ is the relative viscosity, and *η*_sp_ is the specific viscosity. The average molecular weight *M*_*η*_ of the polymer is calculated according to the Mark–Houwink equation:

4 [*n*] = *KM*_*η*_^*α*^

where *K* = 0.0123 ml g^−1^; *α* = 0.83.

Using an indium-calibrated DSC (Discovery DSC 25, TA Instruments), *T*_g_ and the enthalpy loss (Δ*H*_a_) due to physical aging were measured. Approximately 8 mg of the polymer sample was placed in an aluminum pan and heated at a rate of 10 °C min^−1^ to 200 °C, where it was isothermally held for 3 minutes. Then, it was cooled at a rate of 10 °C min^−1^ to 20 °C, and subsequently, heated again at a rate of 10 °C min^−1^ to 200 °C. For dry samples, the inflection point in the transition zone during the second heating process was taken as the *T*_g_. In contrast, for samples that absorbed water due to hygrothermal aging, to prevent the evaporation of water from affecting the test results, the inflection point in the transition zone during the first heating process was selected as the *T*_g_. The enthalpy loss (Δ*H*_a_) at a specified aging temperature (*T*_aging_) over the corresponding time (*t*) was calculated as follows:5
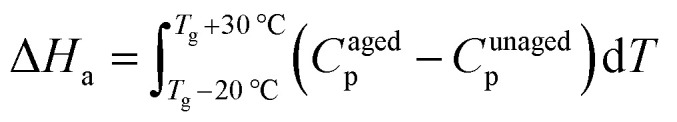
where *C*^aged^_p_ and *C*^unaged^_p_ respectively represent the heat capacity curves of the aged (first heating cycle) and unaged (second heating cycle) samples. To eliminate the influence of moisture absorption on the *T*_g_ test, the samples were vacuum-dried at 50 °C for 24 h before testing.

The refractive index *n*_d_ of the copolymer PC was measured at a wavelength of 589 nm using an Abbe refractometer (DR-M2).

The PC films were cut into dimensions of 8 × 10 × 0.08 cm and soaked in water at 80 °C to conduct water absorption and hygrothermal aging tests. The formula for calculating water absorption rate was as follows:6
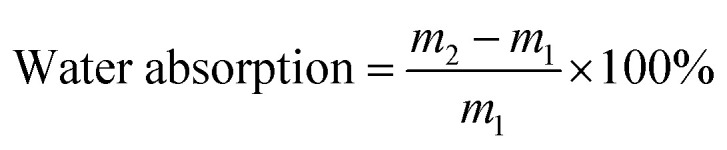
where *m*_1_ and *m*_2_ represent the mass of the film before soaking and after soaking for a certain period of time, respectively. Each sample group was tested three times, and the average value was taken as the final result.

The tensile strength and elongation at break of the samples are tested at room temperature using an INSTRON 3365 universal testing machine. Square specimens (75 × 4 × 0.8 mm) were prepared using the solvent casting method described above. The tensile rate was set to 10 mm min^−1^. Each sample was tested at least 5 times, and error bar graphs were plotted.

## Results and discussion

### Molecular structure and physical properties of copolymerized PC

**Fig. 1:**
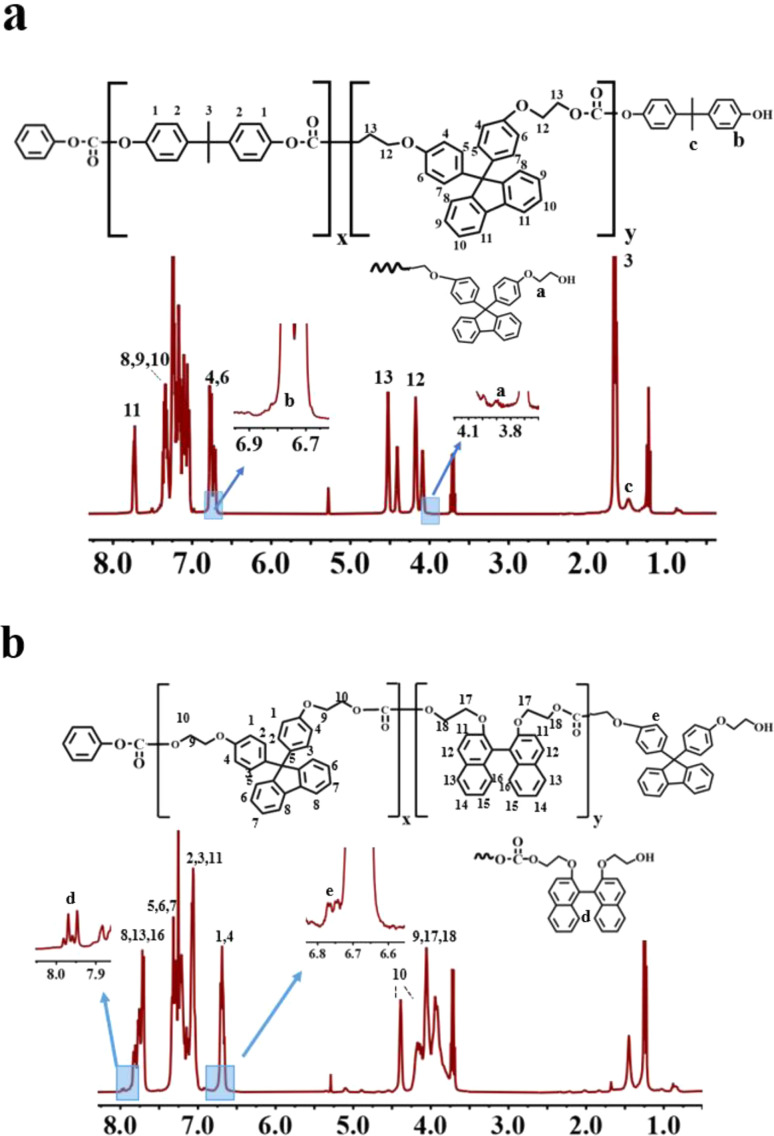
^1^H-NMR spectrum of (a) BPA/BPEF(50/50)-PC and (b) BNE/BPEF(49/51)-PC.

Fig. 1a and b show the ^1^H-NMR spectra for BPA/BPEF (molar ratio 50/50) and BNE/BPEF (molar ratio 49/51), respectively. From Fig. 1a, it is observed that the peak a′, representing the monomeric structure of BPEF, shifts from 4.0 ppm to 4.1 ppm, corresponding to peak number 12 in the main chain structure. Simultaneously, the peak c′, indicative of the BPA monomeric structure, shifts from 1.5 ppm to 1.6 ppm, identified as peak number 3. These shifts signifies the successful copolymerization. The figure presents the a′ peak representing the end groups of BPEF. However, the accurate calculation of the BPA terminal group content is hindered by the influence of peak number 3 on the c′ peak. Additionally, the b′ peak is obscured by peaks 4 and 6. The molar content of the terminal hydroxyl groups is calculated by subtracting twice the area of peak number 11 from the sum of the areas of peaks 4 and 6 (Table 1). The terminal phenoxy group peak around 7.4 ppm is obscured by peaks 7.3 ppm from the BPEF main chain structure in peaks 8, 9, 10. From Fig. 1b, it is observed that the d’ peak representing BNE shifts from 7.95 ppm to 7.8 ppm, and simultaneously, the e′ peak indicative of BPEF shifts from 6.75 ppm to 6.70 ppm. These shifts indicate the successful copolymerization of the two components. With peaks d′ and e′ representing the terminal hydroxyl groups of BNE and BPEF, respectively, allowing for the calculation of the terminal hydroxyl group molar content (Table 1). The terminal phenoxy group peak around 7.4 ppm is obscured by peaks 5, 6, 7 from the BPEF main chain structure, thus the molar content of the terminal phenoxy group for these copolymers was determined using liquid chromatography methods^23,24^ (Table 1).

**Fig. 2:**
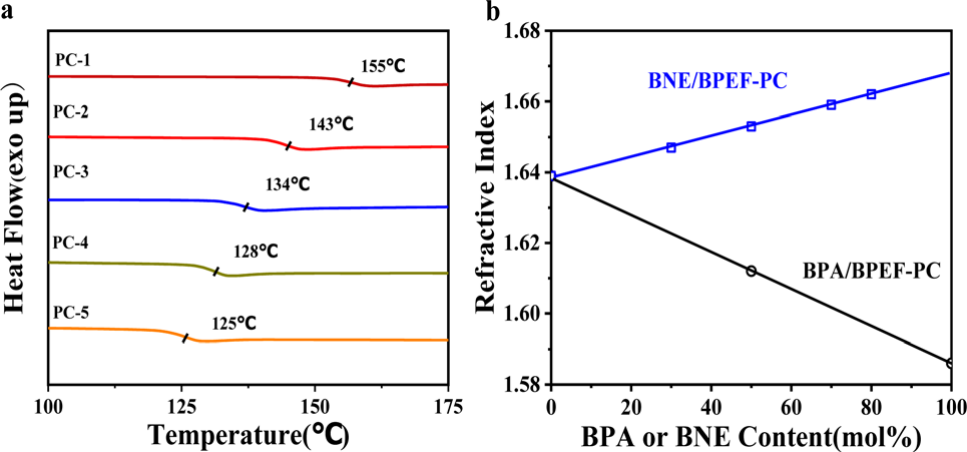
(a) DSC curves for different BNE/BPEF-PC copolymers and (b) BNE or BPA content dependency of refractive index.

Table 1 lists the viscosity-average molecular weight, number-average molecular weight (determined based on terminal groups measurements), *T*_g_, color difference, optical transmittance, refractive index and orientation birefringence values for different copolymer ratios of BPA, BNE and BPEF. By adjusting the copolymer ratio, a series of copolymer PCs was synthesized, with color difference values tested using a UV/visible spectrophotometer that was less than 1, and the *T*_580_, measuring transparency, was found to be 81–83% for films formed by solvent casting, indicating that the sample exhibited significant yellowing and had reduced transparency. As shown in Fig. 2a, for the BNE/BPEF system, the *T*_g_ of the copolymer PC showed a decreasing trend with an increase in BNE content, mainly due to the introduction of the binaphthyl structure of BNE, where the two naphthalene rings positioned adjacently form a non-coplanar conformation due to their large volume, disrupting interchain packing and thus lowering the *T*_g_ value of the polymer.^25^ The refractive index of the BPEF homopolymer PC was 1.639, and the refractive index of the copolymer PC increased with the increase in BNE content, reaching 1.661 when the BNE molar content was 80%, meeting the requirements for high refractive index. After uniaxial stretching at *T*_g_ + 15 °C, the orientation birefringence value Δ*n* at 589 nm was measured using an ellipsometer (M-220), as shown in Table 1.^26^ It was observed that when the copolymer content of BNE is less than 70 mol%, the Δ*n* value is negative, and as the BNE content increases, this value approaches zero. When the BNE content reaches 90 mol%, the Δ*n* value turns positive. Therefore, when the BNE/BPEF copolymer ratio is close to 70/30, it is most likely to achieve near-zero birefringence, at which point the refractive index is 1.659.

### Water absorption behavior

**Fig. 3:**
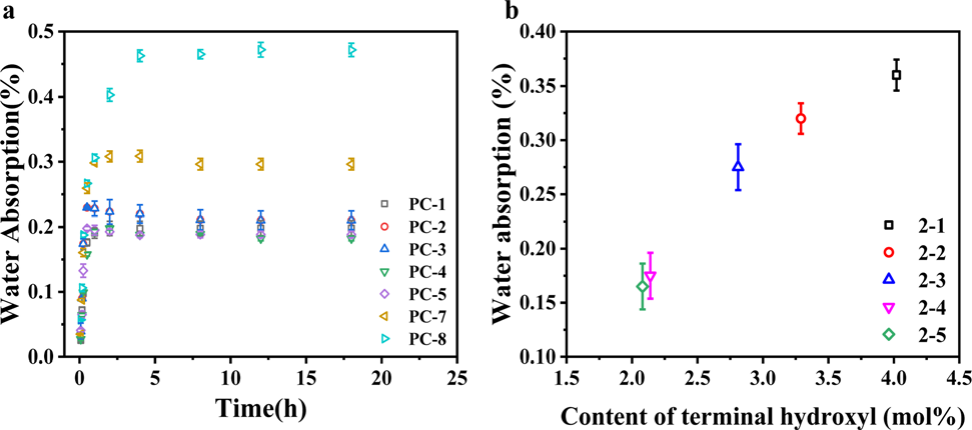
(a) Time dependency of water absorption for PC copolymers immersed in water at 80 °C; (b) influence of terminal hydroxyl content on equilibrium water absorption at 80 °C.

Polycarbonate's susceptibility to water absorption is primarily attributed to its ester bonds, which can affect its performance in practical applications. Fig. 3a delineates the water absorption rates of various PCs following immersion at 80 °C for a predetermined duration. The equilibrium water absorption rate of PC-8 is observed to be 0.46%, congruent with behaviors of BPA-PC in previous reports.^27^ Introducing additional phenyl rings increases volume of the hydrophobic groups within the molecular chain, making it more difficult for water molecules to penetrate the polymer, thus potentially reducing the water absorption rate. Conversely, the presence of ether bonds in the main chain structure, which are prone to absorbing water, may elevate the water absorption rate.^28^ Nevertheless, the experimental results indicate that the hydrophobic effect of the phenyl rings plays a dominant role. The introduction of a 50% molar content of BPEF into PC-7 resulted in a reduction of the equilibrium water absorption rate at 80 °C to approximately 0.30%. It is also observed that despite the different copolymer ratios in PC-1–5, the equilibrium water absorption rates stabilized at 0.20% after 5 h of soaking, suggesting that variations in copolymer ratio do not significantly affect water absorption in this system. However, water absorption is also related to the terminal hydroxyl group content; an increase in terminal hydroxyl groups corresponds to a higher water absorption rate.^29^ The slightly lower terminal hydroxyl content measured by ^1^H-NMR for PC-4 and PC-5 correlates with their lower water absorption rates compared to PC-1–3.

To determine the impact of terminal hydroxyl group content on water absorption, a series of BNE/BPEF(70/30)-PC copolymers, exhibiting varied levels of terminal hydroxyl groups, were synthesized, as delineated in Table 2. Fig. 3b illustrates that the equilibrium water absorption rate increases significantly with the copolymer's terminal hydroxyl content, reaching up to 0.37%. Notably, when the terminal hydroxyl group content is maintained at 2.08 mol%, the equilibrium water absorption rate is constrained to a mere 0.15%. Therefore, to reduce the water absorption rate of copolymer PCs, it is imperative to regulate the terminal hydroxyl group content, ensuring it remains at a judicious level.

**Table 2:** BNE/BPEF(70/30)-PC number-average molecular weight (*M*_n_, determined based on terminal groups measurements), terminal hydroxyl content [OH], terminal phenoxy content [–OPh], viscosity-average molecular weight (*M*_*η*_), and equilibrium water absorption

Samples	*M*_n_ (g mol^−1^)	[–OH] (mol%)	[–OPh] (mol%)	*M*_*η*_ (kg mol^−1^)	Equilibrium water absorption (%)
2-1	16.8	4.04	0.95	26.1	0.37
2-2	21.4	3.29	0.62	30.1	0.33
2-3	19.6	2.52	1.75	26.1	0.26
2-4	22.9	2.14	1.52	33.5	0.16
2-5	18.6	2.08	2.42	26.5	0.15

### Hydrolysis stability

The description provides a comparison of the hydrolytic stability of two types of polycarbonate (PC) materials under a hygrothermal environment. Hydrolysis tests were conducted at 80 °C for various PC samples, measuring the intrinsic viscosity molecular weight at the initial moment and at a specific time, followed by normalization (*M*_*ηt*_/*M*_*η*0_).

**Fig. 4:**
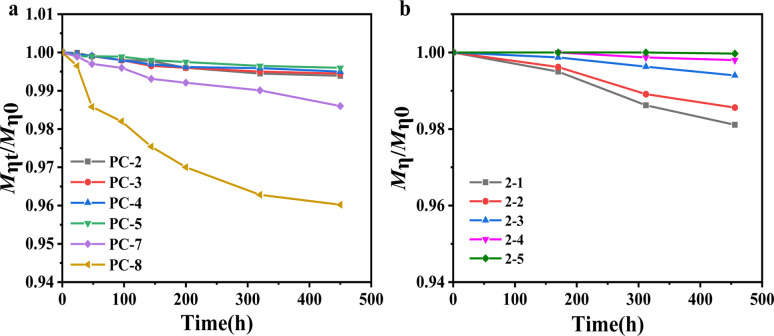
Variation of molecular weight *M*_*η*_ over time for (a) BPA-PC and BNE/BPEF-PC with different BNE/BPEF ratios and (b) BNE/BPEF(70/30)-PC with different contents of the terminal hydroxyl group immersed in 80 °C water.

From Fig. 4a, it is evident that after 450 h of hydrolysis testing, the molecular weight of BNE/BPEF-PC do not show a significant decrease, whereas BPA-PC (PC-8) exhibits a pronounced decrement. From the above results, it can be seen that water absorption is one of the factors influencing hydrolysis. As water serves as an important solvent for the hydrolysis reaction, an increase in its content naturally accelerates the hydrolysis process. In the case of BPA-PC, hydrolysis is primarily associated with the content of terminal phenolic hydroxyl groups. Bisphenol A has a p*K*_a_ value of 9.78, with its terminal phenolic hydroxyl groups exhibiting acidity. The acidic terminal phenolic hydroxyl groups will dissociate H_3_O^+^ in a hygrothermal environment, thereby assailing the ester groups and facilitating hydrolysis. Consequently, increasing the phenoxide end-capping rate is an effective choice. BNE (p*K*_a_ = 13.87) and BPEF (p*K*_a_ = 13.79), with p*K*_a_ values close to that of water (p*K*_a_ = 15.70), cannot dissociate sufficient H_3_O^+^ to promote hydrolysis under a hygrothermal environment.

Fig. 4b portrays the hydrolysis behavior of copolymer PCs with different terminal hydroxyl group contents under 80 °C water. It can be observed that as the terminal hydroxyl group content increases, the hydrolysis becomes more pronounced. When the terminal hydroxyl group content exceeds 4 mol%, the molecular weight undergoes a reduction exceeding 2% after 450 h of hydrolysis; when the terminal hydroxyl group content is about 2 mol%, hydrolysis essentially does not occur. Therefore, to mitigate hydrolysis issues caused by hygrothermal aging, it is necessary to reduce the terminal hydroxyl group content and increase the phenoxide end-capping rate.

### Physical aging in hygrothermal environment

**Fig. 5:**
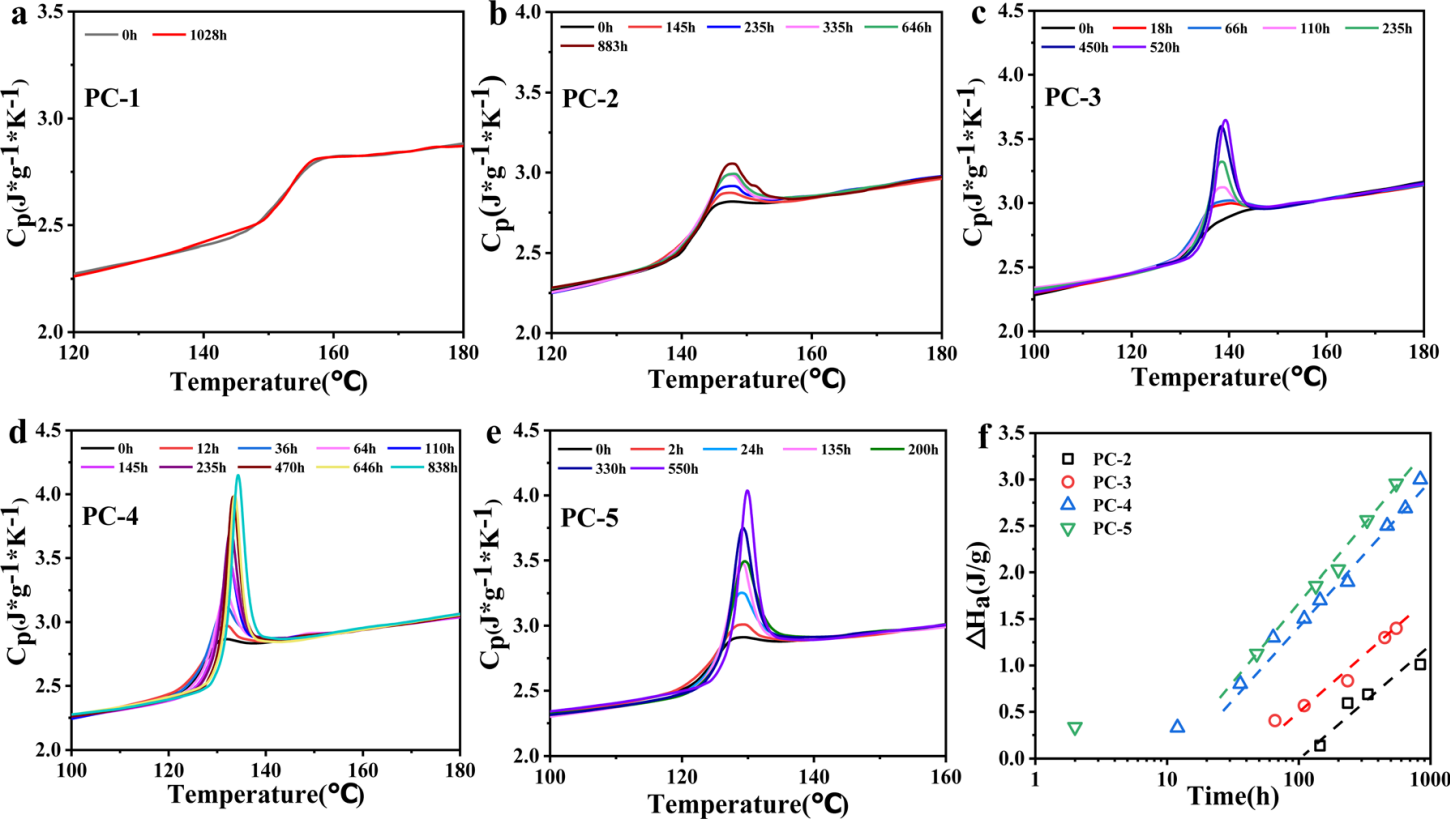
(a–e) DSC curves for PC-1–5 after hygrothermal aging at 80 °C for different time; (f) variation of enthalpy loss over hygrothermal aging time at 80 °C.

In the previous section, it is explained that BNE/BPEF-PC with a high phenoxide end-capping shows excellent hydrolysis resistance. Experimental results also found that some PC copolymers tend to become brittle after aging at 80 °C in a hygrothermal environment, which is believed to be due to physical aging occurring during the hygrothermal aging test.^16,18^ DSC is commonly used to quantitatively measure the enthalpy loss (Δ*H*_a_) to indicate the degree of physical aging, as shown in Fig. 5.

The DSC results show that for PC-2–5, with the progression of time, different degrees of overshoot peaks appear near *T*_g_, indicating varying degrees of physical aging. As the copolymer ratio of BNE in BNE/BPEF-PC increases, *T*_g_ decreases, and the overshoot peaks become more pronounced. Conversely, PC-1, with a *T*_g_ of 155 °C, does not exhibit any significant overshoot peak after 1028 h of aging at 80 °C, suggesting that PC-1 does not undergo physical aging at 80 °C. Fig. 5f shows the change in enthalpy loss of PC-2 to PC-5 with logarithmic time. PC-2 with a *T*_g_ of 143 °C does not show significant overshoot peaks within 100 h of hygrothermal aging at 80 °C. However, after a long duration (145 h) of aging, small overshoot peaks gradually appear, with a Δ*H*_a_ value of only 0.98 J g^−1^ after 883 h; PCs 3–5 exhibit significant overshoot peaks within 18 h of aging, and at the same aging time, the components with lower *T*_g_ values produce more pronounced overshoot peaks. Among them, PC-5 reaches a Δ*H*_a_ of 3.0 J g^−1^ after 550 h of aging, indicating severe physical aging. The Fig. 5f shows that the increase behavior of enthalpy loss can be obtained using a simple linear fit over a specific range.^18^

**Fig. 6:**
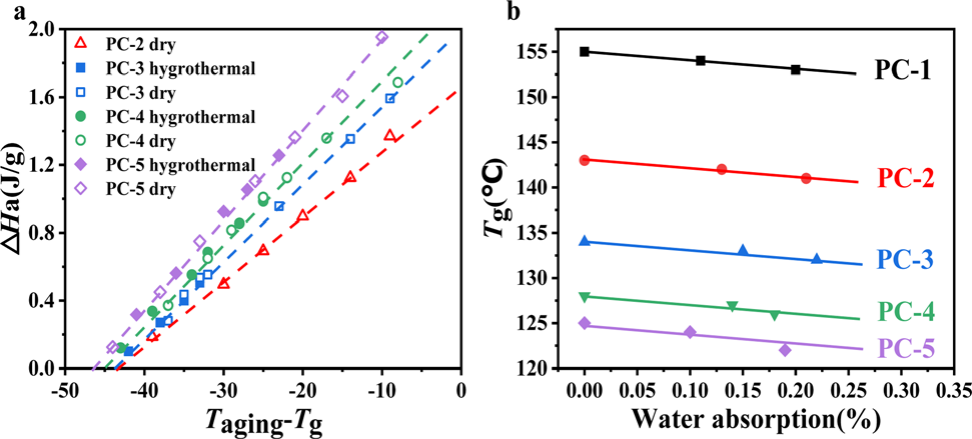
(a) Enthalpy loss of PCs at different aging temperatures for 10 h and (b) the relationship between *T*_g_ and water absorption of PCs.

To further investigate the window in which physical aging occurs for various PCs, we measured the physical aging behavior of PC samples in both dry and hygrothermal environments at different temperatures for 10 h each, as illustrated in Fig. 6. From Fig. 6a, the physical aging windows for PCs in both dry and hygrothermal environments can be inferred by linearly fitting the obtained data points to the *x*-axis intersection. It is observed that the lowest temperatures at which physical aging occurs for PC-2–5 are 45 ± 1 °C below *T*_g_, indicating that significant physical aging phenomena can occur within a short time at temperatures ranging from *T*_g_-45 to *T*_g_. It should be noted that the degree of aging varies with different copolymer ratios, primarily indicating that with an increase in BNE content, the higher the Δ*H*_a_ value at the normalized temperature (*T*_aging_ − *T*_g_), reflecting a more pronounced aging degree. Physical aging essentially reflects the mobility of molecular structures. In BPEF structures, the four benzene rings are compactly arranged due to van der Waals forces, resulting in poorer molecular mobility. In contrast, BNE's linked naphthalene ring structure, with its large naphthyl groups, prevents the polymer chains from packing closely together,^30^ making the overall molecule relatively loose. This facilitates greater movement at temperatures nearing *T*_g_, thereby enabling the system to evolve more rapidly towards equilibrium.

Fig. 6b shows the trend of *T*_g_ changes for BNE/BPEF-PC after water absorption, revealing that due to the water absorption rate being only approximately 0.20%, the induced decrease in *T*_g_ is minimal, only 2–3 °C. Comparing the enthalpy losses in dry and hygrothermal environments, it is found that at equivalent temperatures, the hygrothermal environment leads to water absorption by the PC copolymer, resulting in a slight decrease in *T*_g_ and a marginally higher degree of physical aging compared to the dry environment. Nevertheless, the enthalpy losses observed in both dry and hygrothermal conditions can be normalized (*T*_aging_ − *T*_g_) to a singular linear relationship, as shown in Fig. 6a, illustrating the close correlation between the degree of physical aging and *T*_g_.

### Effect of hygrothermal aging on mechanical properties

**Fig. 7:**
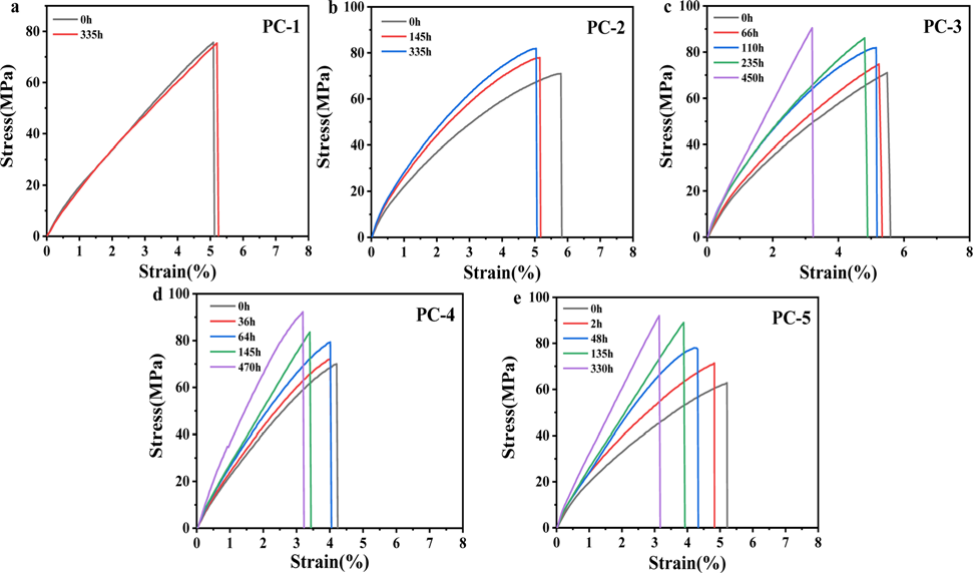
(a–e) Stress–strain curves for various PCs after hygrothermal aging at 80 °C for different times.

Fig. 7 presents the mechanical property of PCs after hygrothermal aging at 80 °C. To eliminate the impact of water plasticization on mechanical properties while precluding further physical aging, the aged samples were vacuum-dried at 50 °C for 24 h to expunge absorbed moisture. It can be observed that with the increase in aging time, the tensile strength of PCs gradually increases, accompanied by a reduction in the elongation at break. It is found that the increase in tensile strength is most significant for PC-5, which is caused by physical aging in a hygrothermal environment. Physical aging induces a denser stacking of molecular chains and a diminution in free volume, culminating in enhanced tensile strength.

**Fig. 8:**
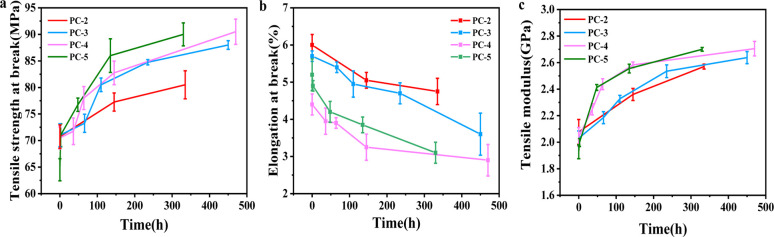
(a) Tensile strength at break *σ*_b_, (b) elongation at break and (c) tensile modulus *E* for various PCs after hygrothermal aging at 80 °C for different time.

Fig. 8a, b and c respectively show the increase in tensile strength at break *σ*_b_ and tensile modulus *E* as aging time extends, along with elongation at break decrease, which is consistent with the trend of physical aging changes in polymers reported previously.^31,32^ It is imperative to acknowledge that the most significant increments in tensile strength and modulus are observed for PC-5, correlating with its heightened degree of physical aging over the same aging period.

### Effect of hygrothermal aging on the transparency of PCs

**Fig. 9:**
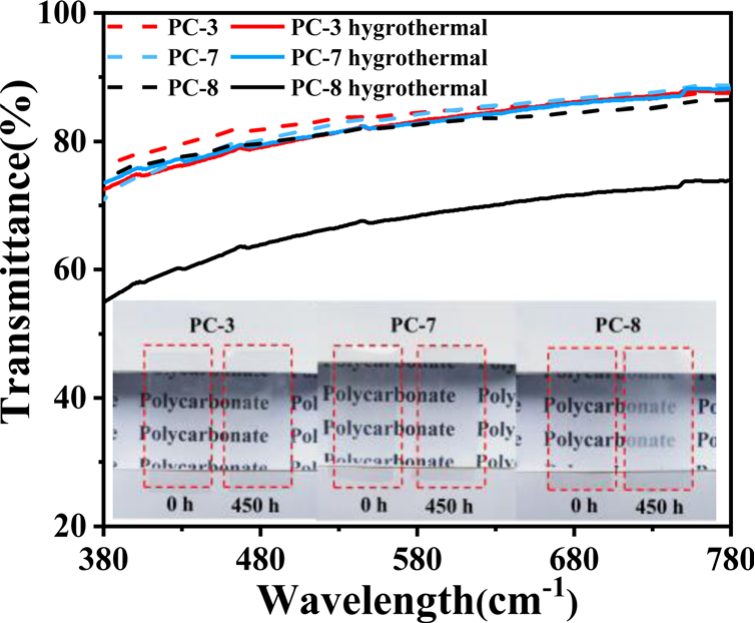
The changes in transparency of PCs before and after 450 hours of hygrothermal aging at 80 °C.

The change in transparency of PC films before and after undergoing 450 hours of hygrothermal aging at 80 °C was tested as shown in Fig. 9. It can be observed that PC-3 and PC-7 still maintain high transparency after hygrothermal aging. However, due to its slightly higher water absorption, water molecules may interact with the polycarbonate molecules to form microstructures or interfaces, which could interfere with light transmission.^33^ Additionally, the penetration of water molecules into the polycarbonate can cause refraction of light between them, thereby affecting transparency. Fig. 4a demonstrates that PC-8 undergoes significant molecular weight reduction after prolonged hygrothermal aging, resulting in the presence of a large amount of low-molecular-weight substances within BPA-PC, which may also contribute to decreased transparency. On the other hand, PC-3(BNE/BPEF-PC) maintains high hydrolytic stability and transparency even after the hygrothermal aging test, further highlighting its excellent optical stability.

## Conclusions

This study, by copolymerizing BNE with BPEF and adjusting the copolymer ratio of BNE to BPEF to 70/30, not only increase the refractive index to 1.659 but also achieve a birefringence value close to zero. The investigation reveals that the incorporation of dihydroxy monomers with elevated p*K*_a_ values for copolymerization significantly enhanced the hydrolytic stability during hygrothermal aging tests compared to BPA-PC. This suggests that the augmentation of the monomers' p*K*_a_ value serves as an effective strategy to suppress hydrolysis.

Furthermore, the research identify that copolymerized polycarbonates undergo physical aging in an 80 °C hygrothermal environment, with the physical aging window of BNE/BPEF-PC delineated roughly from *T*_g_-45 °C to *T*_g_. The introduction of water causes a reduction in *T*_g_, yet the enthalpy reduction across both dry and hygrothermal conditions can be standardized to a linear relationship by the differential between the aging temperature and *T*_g_. This delineates a strong correlation between the extent of physical aging and *T*_g_. As the content of BNE increases, the degree of physical aging becomes more pronounced. Mechanical performance testing of physically aged samples reveal that more pronounced physical aging corresponded to significant increases in tensile modulus and fracture strength, underscoring the direct impact of physical aging on the mechanical properties of the material. The transparency test shows that the optical transparency of BNE/BPEF-PC shows great stability before and after hygrothermal aging.

## Conflicts of interest

There are no conflicts to declare.

## Supplementary Material

RA-014-D4RA03378G-s001
