# Supplementary material for: Synthesis and hygrothermal aging of polycarbonates containing a bisphenol fluorene moiety
Source: RSC Adv. 2024 Jun 3;14(25):17771–9. doi: 10.1039/d4ra03378g (PMC11145624; doi:10.1039/d4ra03378g)
Supplement: RA-014-D4RA03378G-s001 [file RA-014-D4RA03378G-s001.pdf]

Electronic Supplementary Material (ESI) for RSC Advances.
This journal is © The Royal Society of Chemistry 2024

# Synthesis and Hygrothermal Aging of Polycarbonates Containing Bisphenol Fluorene Moiety

Tao Lu, Wei Fang, Qian Zhou, Mengjuan Liu, Guozhang Wu*

East China University of Science and Technology, Shanghai 200237, China.

*Corresponding author. Email: 1175003800@qq.com.

**Characterization**

The copolymer ratio of BPA/BPEF and BNE/BPEF was calculated based on the integration area of relevant peaks ($A$, $B$) in the $^1$H-NMR spectrum using specific formulas:

$$\frac{\text{Raito of BPA}}{\text{Raito of BPEF}}=\frac{m}{n}=(\frac{1}{6}A_{1.6})/(\frac{1}{2}A_{7.7}) \tag{1}$$

$$\frac{\text{Raito of BNE}}{\text{Raito of BPEF}}=\frac{y}{x}=(B_{7.6}-\frac{1}{2}B_{6.6})/B_{6.6} \tag{2}$$

where $A_{1.6}$ and $A_{7.7}$ refer to the peak areas of protons at 1.6ppm (peak 3) and 7.7ppm (peak 11) respectively in Fig. 1a; $B_{6.6}$ and $B_{7.6}$ refer to the peak areas of H protons at 6.6ppm (peaks 1 and 4) and 7.6ppm (peaks 8, 13, and 16) respectively in Fig. 1b; m and n represent the copolymer ratio of BPA to BPEF; x and y represent the copolymer ratio of BPEF to BNE, respectively. The formula for calculating the molar content of terminal hydroxyl groups ([-OH]) is as follows:

$$[\text{-OH}]_{\text{BPA/BPEF-PC}}=\frac{[\text{total number of -OH}]}{[\text{number of structural units}]}=\left\{\frac{1}{2}(A_{6.7}-2A_{7.3})+\frac{1}{2}A_{3.9}\right\}/(\frac{1}{6}\frac{m}{m+n}A_{1.6}+\frac{1}{2}\frac{n}{m+n}A_{7.3})\times 100\% \tag{3}$$

$$[\text{-OH}]_{\text{BNE/BPEF-PC}}=\frac{[\text{total number of -OH}]}{[\text{number of structural units}]}=(\frac{1}{2}B_{7.9}+\frac{1}{4}B_{6.7})/(\frac{1}{2}\frac{x}{x+y}B_{7.6}+\frac{1}{4}\frac{y}{x+y}B_{7.6})\times 100\% \tag{4}$$

where $A_{3.9}$, $A_{6.7}$, and $A_{7.7}$ respectively represent the peak areas of protons at 3.9 ppm (peak a), 6.7 ppm (peaks 4 and 6), and 7.3 ppm (peak 11) in Fig. 1a; $B_{7.95}$ and $B_{6.75}$ respectively represent the peak areas of H protons at 7.95 ppm (peak c) and 6.75 ppm (peak d) in Fig. 1b.

The molar content of phenoxyl groups was calculated by the following formula:

$$[\text{-OPh}]_{\text{BPA/BPEF-PC}}=\frac{c_{\text{PhOH}}}{94}/(\frac{m}{m+n}\frac{c_{\text{BPA}}}{228}+\frac{n}{m+n}\frac{c_{\text{BPEF}}}{438})\times 100\% \tag{5}$$

$$[\text{-OPh}]_{\text{BNE/BPEF-PC}}=\frac{c_{\text{PhOH}}}{94}/(\frac{x}{x+y}\frac{c_{\text{BPEF}}}{438}+\frac{y}{x+y}\frac{c_{\text{BNE}}}{374})\times 100\% \tag{6}$$

where $c_{PhOH}$, $c_{BPEF}$, and $c_{BNE}$ represent the concentrations of PhOH, BPEF, and BNE, respectively.

Using a UV/Visible spectrophotometer (UV1900, Youke Instrument) to measure the transmittance and color difference of copolymer PC. The light transmittance of the copolymer film in the visible light region is tested, with air as the blank reference, and the transmittance of the sample at 580 nm was recorded as $T_{580}$ (%). When measuring the color difference of the copolymer polycarbonate, the samples were dissolved in chloroform to prepare a solution with a concentration of 0.01 g/mL. The color difference $\Delta C$ was calculated as follows:

$$\Delta C = \left(\left|\frac{1}{3} - \frac{T_{600}}{T}\right| + \left|\frac{1}{3} - \frac{T_{555}}{T}\right| + \left|\frac{1}{3} - \frac{T_{445}}{T}\right|\right) \times 100\% \tag{7}$$

where $T = T_{600} + T_{555} + T_{445}$, $T_{600}$, $T_{555}$ and $T_{445}$ refers to the transmittance of the sample at wavelengths of 600 nm, 555 nm, and 445 nm.
